# Combination of Anlotinib and Celecoxib for the Treatment of Abdominal Desmoid Tumor: A Case Report and Literature Review

**DOI:** 10.3389/fonc.2021.830672

**Published:** 2022-01-13

**Authors:** Jianzheng Wang, Hongle Li, Hui Wang, Qingli Li, Xuanye Bai, Huifang Lv, Caiyun Nie, Beibei Chen, Weifeng Xu, Shuiping Tu, Xiaobing Chen

**Affiliations:** ^1^ Department of Medical Oncology, Affiliated Cancer Hospital of Zhengzhou University, Henan Cancer Hospital, Zhengzhou, China; ^2^ Department of Molecular Pathology, Affiliated Cancer Hospital of Zhengzhou University, Henan Cancer Hospital, Zhengzhou, China; ^3^ Department of Endoscope Center, Affiliated Cancer Hospital of Zhengzhou University, Henan Cancer Hospital, Zhengzhou, China; ^4^ Department of Oncology, Renji Hospital, School of Medicine, Shanghai Jiaotong University, Shanghai, China; ^5^ Department of Pathology, Affiliated Cancer Hospital of Zhengzhou University, Henan Cancer Hospital, Zhengzhou, Henan, China

**Keywords:** desmoid tumors, anlotinib, celecoxib, targeted therapy, NSAIDs

## Abstract

Desmoid tumor is a rare disease, which is histologically characterized by local invasion, monoclonality, and fibroblast proliferation; and clinically characterized by a variable and often unpredictable course. The treatment of desmoid tumor is mainly surgical resection, but the recurrence rate is high. In recent years, a variety of treatment methods, including endocrine therapy, surgery, radiotherapy, chemotherapy, non-steroidal anti-inflammatory drugs, targeted drugs, interferon and more, have been used and achieved certain curative effects. In addition, in view of the inertia characteristics of desmoid tumor, observation is also a first-line scheme recommended by multiple guidelines. In the past, the research progress of targeted therapy for desmoid tumor is relatively slow and the curative effect is limited. Thus, targeted therapy is usually used as a remedial treatment after the failure of other conventional treatment methods. However, in recent years, with the rapid progress in the basic research of targeted therapy, some new targeted drugs are increasingly used for the clinical treatment of desmoid tumor and have achieved good results. Herein, we described a patient with aggressive fibromatosis in the abdominal cavity. Following a combined treatment using anlotinib and celecoxib, the patient achieved a partial response with mild toxicity. Simultaneously, the patient’s pain symptoms completely disappeared. This case indicates that the combination of anlotinib and NSAIDs could be an effective treatment for desmoid tumor.

## Background

Desmoid tumors (DTs), also known as aggressive fibromatosis, are local tumors of mesenchymal origin that can cause significant morbidity due to their infiltrating nature (1, 2). DTs are fibroblast clonal proliferative diseases originated from deep soft tissue. Most of them are found in connective tissues in muscles and fascia or aponeurosis. DT often infiltrates into adjacent muscle tissue or adipose tissue. It is usually easy to relapse after surgical resection, but in some cases, the disease can be stable and subside by itself (3). DTs include sporadic fibroma and familial adenomatous polyposis (FAP) associated fibroma. Most DTs are sporadic and are mainly caused by abnormalities in the Wnt/β-catenin signaling pathway, usually related to somatic β-catenin gene mutations (4, 5). At present, the treatment of DTs includes active monitoring, surgery, radiotherapy, chemotherapy, targeted therapy, endocrine and non-steroidal drug therapy (6). In the past, the research progress of targeted therapy for DTs is relatively slow and the curative effect is limited. Therefore, targeted therapy is often used as remedial treatment after the failure of other conventional treatment methods. However, in recent years, with the rapid progress of basic research on targeted therapy, some new targeted drugs, such as such as Imatinib (7–9), Pazopanib (10) and Sorafenib, are used in the clinical treatment of DTs and have achieved good curative effect (11).

Anlotinib is a small molecule multi-target tyrosine kinase inhibitor, which can effectively inhibit vascular endothelial growth factor receptor (VEGFR), platelet-derived growth factor receptor (PDGFR), fibroblast growth factor receptor (FGFR), stem cell growth factor receptor (c-Kit) and others, and has the effect of anti-tumor angiogenesis and inhibiting tumor growth (12). Anlotinib has shown encouraging anti-tumor effects and acceptable toxicity in advanced lung cancer and soft tissue sarcoma (13–15). However, the role of anlotinib in the treatment of DTs remains unknown. So far, there is no clinical study on using anlotinib for the treatment of DTs.

We have recently encountered a rare case of isolated aggressive fibromatosis located in the abdominal cavity. Our team innovatively used the combination of anlotinib and celecoxib for the treatment. The patient’s tumor was significantly reduced and the progression-free survival (PFS) exceeded 18 months. This is the first case of successful treatment of DT using the combination of anlotinib and non-steroidal anti-inflammatory drugs (NSAIDs), which has not been previously reported.

## Case Presentation

**Figure 1:**
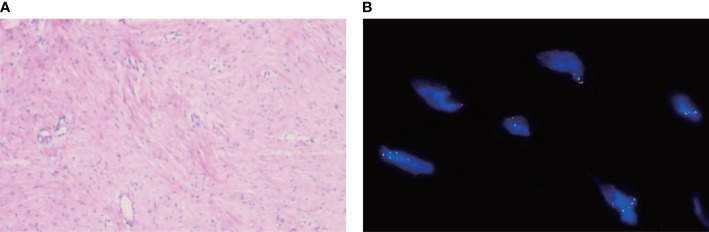
**(A)** HE staining showing the fibromatosis. **(B)** Fluorescence *in situ* hybridization(FISH) detection:3p22/3 = 0.72,CTNNB1 gene deletion. Probe type: CSP3/GSP CTNNB1 (3p22).

**Figure 2:**
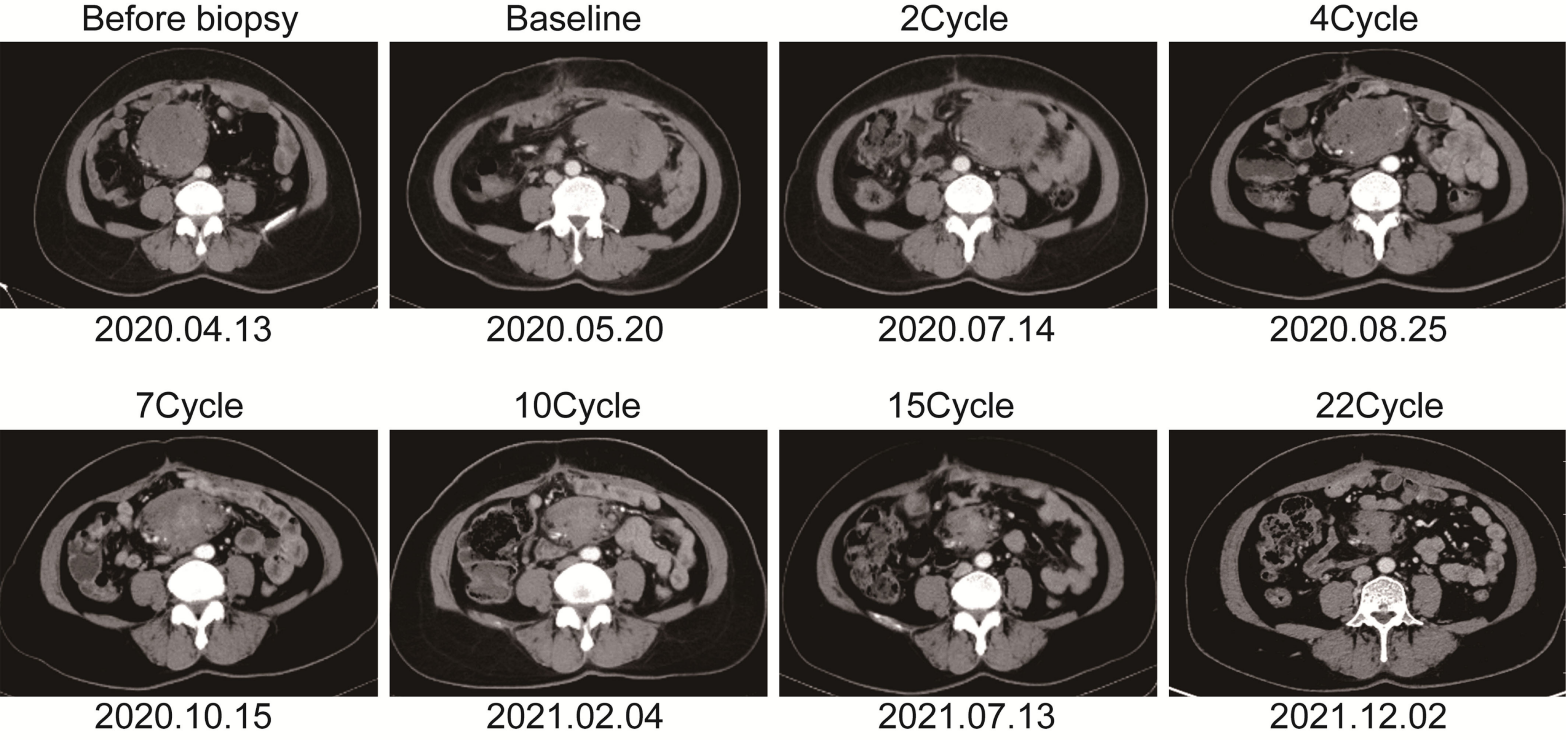
The soft tissue mass in the left lower abdomen was scanned with enhanced computed tomography at different time points.

**Figure 3:**
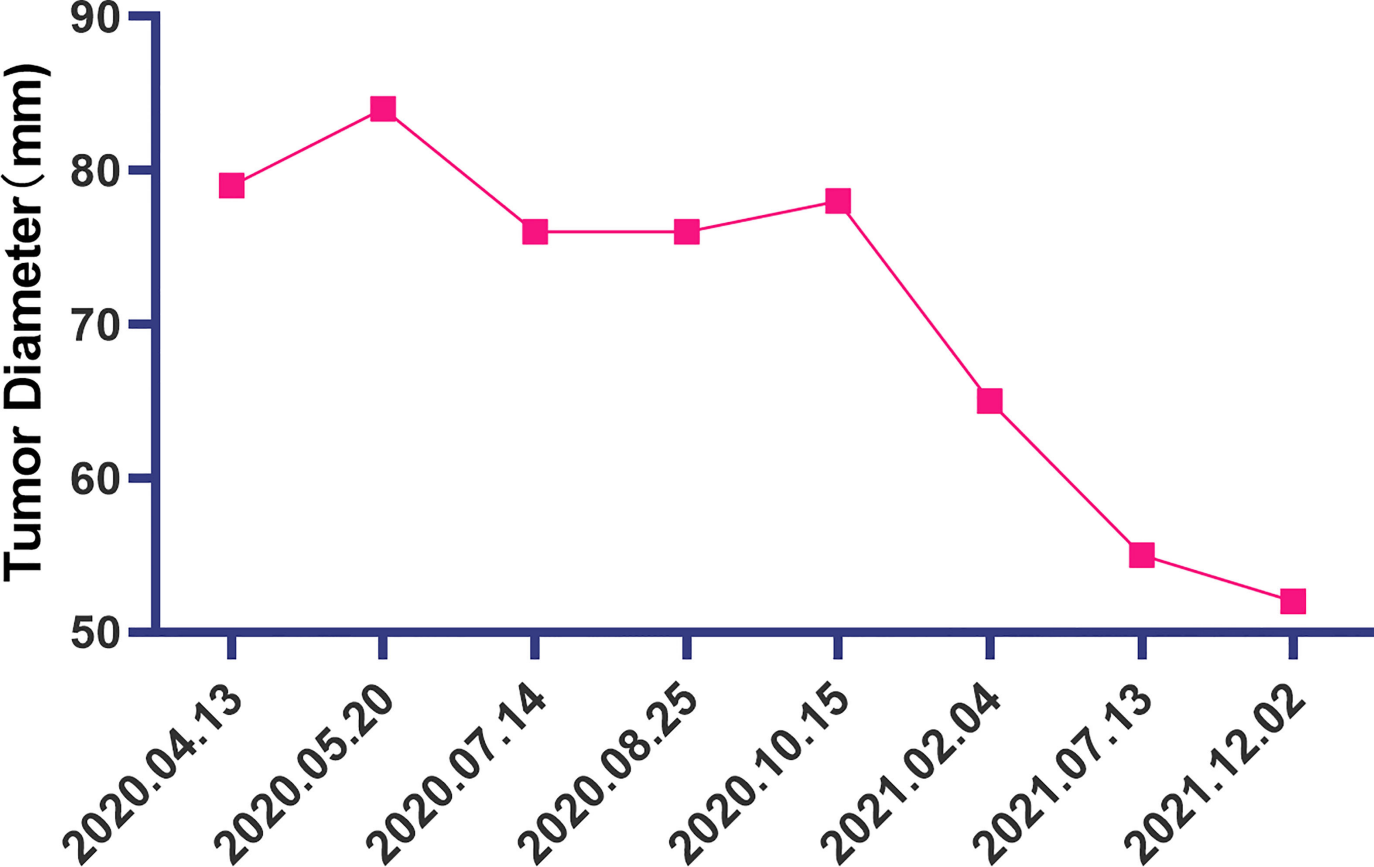
The change of the diameter of the soft tissue mass in the left lower abdomen at different time points.

**Figure 4:**
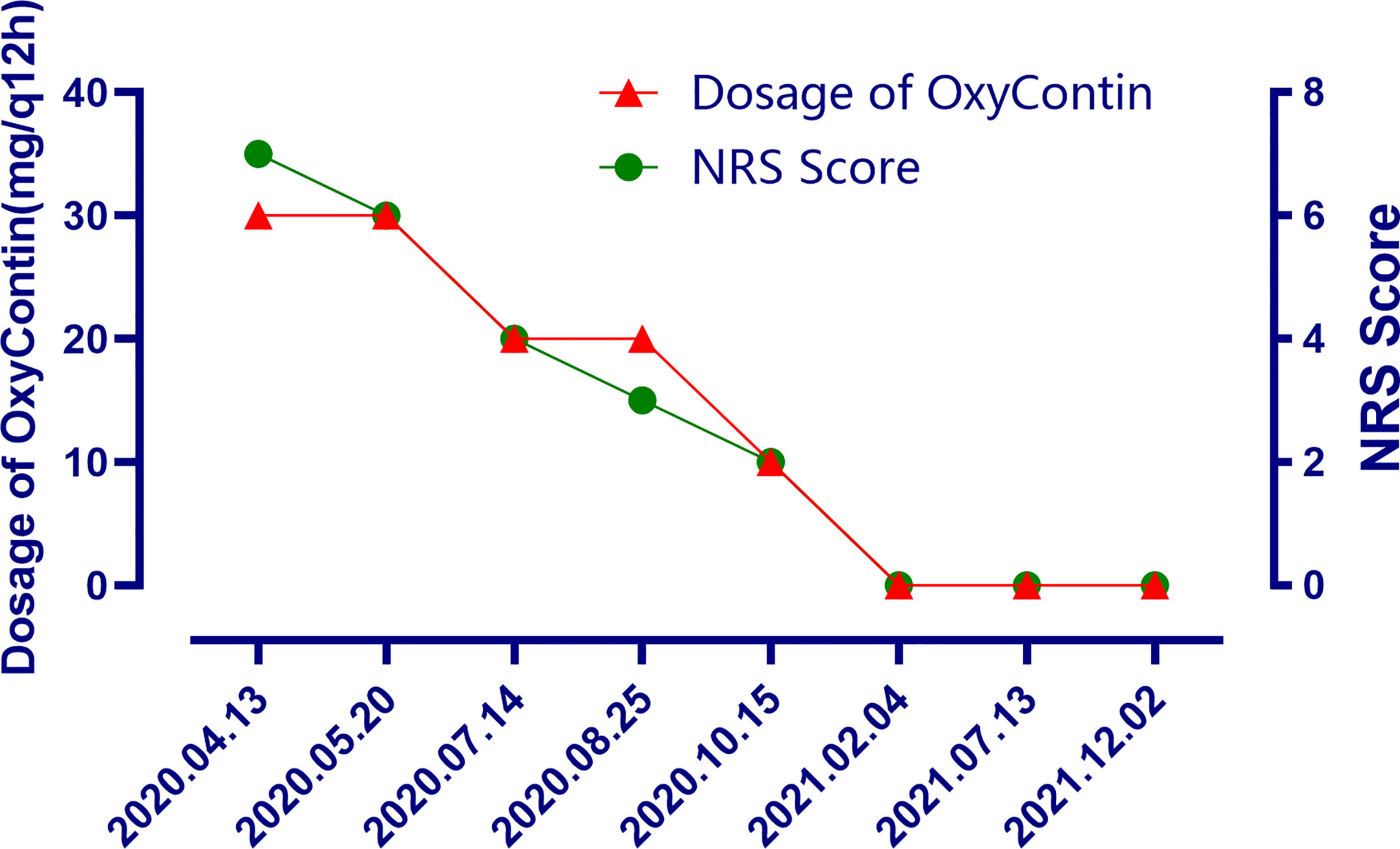
The changes of the dosage of OxyContin and the changes of NRS scores after treatment at different time points.

The patient is a 50 years old woman who had accidentally palpated a hard mass in the lower abdomen one and a half years ago. The mass had unclear boundaries and the patient had gradually worsening pain. The numerical rating scale (NRS) score was 7 points. A color Doppler ultrasound examination at the local hospital (2020-04-12) showed that there was an inhomogeneous echo mass about 9.5cm*7.7cm*6.8cm in the right front of the abdominal aorta in the upper mid-abdomen. She was admitted to Henan Cancer Hospital and an enhanced CT examination (2020-04-14) showed that there is a lump in the lower part of the abdomen and the upper part of the pelvis. The exploratory laparotomy was performed under general anesthesia on 2020-04-29. During the surgery, the size of the tumor was about 9cm*7cm*6cm, which invades the mesentery and causes mesangial contracture. The tumor envelops the main branches of the upper mesenteric blood vessels and is closely related to the blood vessels. Abdominal mass biopsy was performed after communicating with the patient’s family. Postoperative pathology (2020-05-18) showed aggressive fibromatosis. Fluorescence *in situ* hybridization (FISH) detection:3p22/3 = 0.72,CTNNB1 gene deletion. Probe type: CSP3/GSP CTNNB1 (3p22) (**Figure 1**). Immunohistochemical staining was performed and showed CK(-), CD34(vascular+), SOX-10(-), Bcl-2 (-),β- catenin(+), SMA(-), Desmin(-), Ki-67 (about 1%), CD117 (-), Dog-1 (-), ER-, PR- (**Supplementary Figure 1**). After the surgery, the patient’s NRS score was 6 points, and was given OxyContin 30 mg po q12h. We performed CT examination again before treatment as a baseline and the enhanced CT (2020-05-20) examination showed: 1. There is a soft tissue mass in the left lower abdominal cavity, with unclear borders, uneven enhancement, and unclear demarcation from the adjacent intestine. The larger section is about 84×65mm, and the surrounding fat gaps are blurred. We explain to the patient’s family that there is currently no standard treatment plan for aggressive fibromatosis, and anti-vascular targeted therapy combined with NSAIDs can be selected. The specific treatment regimen was: anlotinib: 10mg po bid d1-14, q21d and celecoxib: 0.1g po bid. From May 28, 2020 to December 02, 2021, anlotinib combined with celecoxib regimen was given for 22 cycles. The abdominal mass was significantly reduced after treatment (**Figure 2**). The treatment efficacy reached PR (partial response) (**Figure 3**), and the NRS score (2020-10-15) was 0 points (**Figure 4**). At present, the patient is generally in good condition, and the NRS score is 0 point. The patient is expected to obtain long-term survival with the continuation of treatment using anlotinib in combination with celecoxib.

## Discussion

DT is a rare soft tissue tumor with a low incidence, which can occur in all parts of the body (16). The World Health Organization (WHO) defines invasive fibromatosis as clonal fibroblastic proliferative tumor formed in deep soft tissue, which is characterized by invasive growth, local recurrence and no distant metastasis (17, 18). The proximal and abdominal parts of the upper and lower limbs are the most common sites. The diagnosis mainly depends on pathological examination. At present, comprehensive treatment including surgery is the main treatment mode for invasive fibroma. Imaging examination is of great significance for monitoring tumor growth.

The etiology of invasive fibromatosis is not clear, which may be due to the combined effect of genetic, endocrine and physical factors, leading to the defect of connective tissue growth regulation. Invasive fibromatosis patients with Gardner syndrome are familial, suggesting that the disease might has a genetic basis (19). It often occurs in pregnant or post-pregnant women, suggesting that endocrine factors may be involved in tumor growth. Thus, estrogen receptor blockers and aromatase inhibitors are often used for its clinical treatment (20, 21).

At present, the treatment of DTs mainly includes active monitoring, surgery, radiotherapy, chemotherapy, targeted therapy and hormonal therapy. However, due to the lack of a comprehensive understanding of the pathogenesis of DTs and their natural evolution history, it is difficult to determine which types of DTs will not progress, and which DTs patients need active treatment. Studies have shown that the 5-year progression-free survival rate of asymptomatic DTs patients with active monitoring is 50% (22).

At present, drugs for targeted therapy of DTs mainly include tyrosine kinase inhibitors (TKIs), Wnt/β-catenin inhibitors and γ-secretase inhibitors. TKIs are one of the effective treatments for DTs. Clinically effective TKIs include imatinib, sorafenib, sunitinib, pazopani and anlotinib. But due to the low incidence of DTs, there is a lack of prospective studies with large number cases. Most of the current studies are retrospective. Imatinib is often used for remedial treatment of DTs after failure of other treatments. Kasper et al. (8) have shown that imatinib can inhibit tumor progression in DTs patients with Response Evaluation Criteria in Solid Tumours (RECIST) progress. For DTs patients with RECIST progress, the progression arrest rate (PAR) at 6 months after imatinib treatment is 65%.Penel et al. (7) showed that the PARs of patients with RECIST progression DTs and recurrent DTs at 6, 9 and 12 months after imatinib treatment were 80%, 69% and 67%, respectively. Sorafenib is one of the most studied TKIs drugs. It was found that in patients with progressive, refractory or symptomatic DTs, sorafenib could significantly prolong PFS and had long-term effects. The two-year PFS was 81% in the sorafenib-treatment group and 36% in the placebo group. The objective response rate (ORR) was 33% and 20%, respectively, in the two groups. In patients with effective treatment of DTs, the median time for sorafenib group to reach the RECIST efficacy evaluation standard was 9.6 months, and the placebo group was 13.3months (11). A phase II prospective study included 19 patients, including 9 sporadic fibromas and 10 FAP-related fibromas, to explore the efficacy of sunitinib on DTs. The results showed that partial response (PR) and stable disease (SD) were 26.3% and 42.1% respectively; the 2-year PFS was 74.7% and the overall survival (OS) was 94.4% (23). A phase II clinical trial found that pazopani showed efficacy for progressive DTs. Compared with methotrexate combined with vincristine, the PFS of pazopani for 6 months was 83.7% and 45.0%, and ORR was 55% and 37%, respectively. The short-term efficacy of pazopani was significantly better than that of the traditional second-line chemotherapy. A retrospective study explored the therapeutic effect of single-agent anlotinib on DTs. Anlotinib treated DTs patients in the extremities had the PFS of 95.2%, 90.5%, and 84.0% at 3, 6, and 12 months, respectively. The disease control rate (DCR) was 86.0% (18/21), and the ORR was 38.1% (8/21). The adverse reactions mainly included hand-foot syndrome, skin pigmentation, menstrual disorder, nausea and diarrhea, but they were all within the acceptable range (24). Although anlotinib has shown a certain effect on DTs, its final therapeutic effect still needs to be further confirmed by large-scale retrospective studies and prospective studies. So far, there is no report on the combination of anlotini and NSAIDs in the treatment of DTs. Our case shows for the first time that the combination of both has a good tumor inhibitory effect. At present, the patient is still in the treatment process, and it is expected to achieve a complete tumor regression effect.

Due to the critical role of Wnt/β-catenin signaling pathway in DTs formation, their inhibitors may be effective in this disease. Wnt/β-catenin inhibitor tegavivint is a new type of targeted drug and is being studied. It shows therapeutic effect on osteosarcoma both *in vitro* and *in vivo* (25). At present, there is no study on the therapeutic effect of tegavivint on DTs, and only one phase I clinical trial (NCT03459469) is recruiting DT patients.

γ-secretase inhibitor (GSI) is another new type of DTs targeted therapy drug. The γ-secretase inhibitor PF-03084014 acts on Notch pathway, interacts with Wnt/APC/β-catenin pathway, and indirectly regulates Wnt/APC/β-catenin pathway, reducing the proportion of cells in S phase and G2-M phase, thereby inhibiting the growth of DTs. However, PF-03084014 cannot cause cell apoptosis. That is, PF-03084014 cannot kill tumor cells. It can only control tumor progression by inhibiting tumor cell proliferation (26). Kummar et al. (27) suggested that PF-03084014 was effective in the treatment of refractory and progressive DTs. Among the 17 patients with DTs enrolled, 16 patients (94%) were evaluated as effective. Five cases (29%) were evaluated as PR, indicating the potential therapeutic effect of PF-03084014 on DTs. Another phase I clinical trial showed that the ORR of patients with DTs treated with PF-03084014 was high (5 out of 7 cases) (28). However, the existing research samples are small, and large sample studies are still needed to confirm the therapeutic effect of PF-03084014 on DTs.

Tyrosine kinase inhibitors are currently widely recognized drugs and are the main treatment when needed. Using targeted therapy, we need to consider the efficacy and also its toxicity. However, this would not be a problem if treatment with long-term toxicities is balanced against the potential benefit. Toxicity is usually assessed based on acute side effects. The emphasis on long-term toxicity is less, and such data of more recent drugs are not available. Tyrosine kinase inhibitor sorafenib is popular at present. However, they may lead to permanent hypertension and/or hypothyroidism (29). This is an issue that cannot be ignored because patients are generally young and have normal life expectancy. There is a characteristic peak age (approximately 30-40 years)for this disease, and we recognize a female predominance (30). Moreover, the long-term safety of these treatments has never been assessed, since these drugs were developed for patients with metastatic cancer and their life expectancy is very limited.

Research on the mechanism of DT indicated that it is characterized by WNT/oncogene pathway alterations triggering COX-2-mediated constitutive coactivation of PDGFR, and may therefore benefit from combined nonsteroidal anti-inflammatory drug and tyrosine kinase inhibitor treatment (31). We look forward to exploring more therapeutic regimens of PDGFR inhibitors combined with COX-2 inhibitors with less side effects. Anlotinib (PDGFR inhibitor) and celecoxib (COX-2 inhibitor) are very promising combinations. However, the optimal combination dose still needs to be explored. Therefore, it is still an open question to choose which treatment is good for drug therapy for DTs. We should clearly share all available and unknown information with patients in order to make the best decision on personalized treatment.

In general, DT is usually not a life-threatening disease. However, due to chronic pain, functional defects, and psychological problems, the quality of life of patients with DTs is generally decline. In particular, pain control should always be a priority whenever pain is present to allow active surveillance also for symptomatic patients as appropriate. There are few existing literature on the evaluation of health-related quality of life (HRQoL) in patients with DTs. We expect that the upcoming clinical trials should take HRQoL, including functional level and symptoms (most importantly, pain) as the endpoint of study. In this case, after the combined treatment of anlotinib and celecoxib, the pain was significantly reduced and the dose of OxyContin was decreased. Although the lesion was only stable in the first few cycles of CT efficacy evaluation, this suggests that the combination of anlotinib and NSAIDs drugs may be a very promising strategy in pain control. However, we cannot completely rule out that NSAIDs drugs play a certain role in enhancing the analgesic effect of opioids.

## Conclusion

Although the management of DTs has undergone significant changes in recent years, there are many areas of controversy. For patients with active monitoring failure and/or postoperative recurrence of DTs, there is an urgent need for drug intervention. Exploring more efficient and low toxic drugs is the future direction, and the quality of life of patients should be the emphasized. As a physician, we should not only pay attention to the quality of life in the near future, but also to its long-term prediction. Our goal should always be to avoid or limit any possible harm. We expect that the combination of anlotinib and NSAIDs will be a potential treatment strategy for DTs in the future.

## Data Availability Statement

The original contributions presented in the study are included in the article/**Supplementary Material**. Further inquiries can be directed to the corresponding author.

## Author Contributions

JW and XC treated the patient. JW, HLL, HFL, CN, BC, and WX collected the data. JW wrote the original draft. HW, XB, QL, and ST analyzed the data and revised the draft. All authors contributed to the article and approved the submitted version.

## Funding

This work was financially supported by the Science and Technique Foundation of Henan Province (No. 202102310121 for JW), the Medical Science and Technology Co-construction Project of Henan Province (No. LHGJ20200167), the 1000 Talents Program of Central plains (No. 204200510023 for XC), and the Sate Key Laboratory of Esophageal Cancer Prevention & Treatment (No. Z2020000X for XC).

## Conflict of Interest

The authors declare that the research was conducted in the absence of any commercial or financial relationships that could be construed as a potential conflict of interest.

## Publisher’s Note

All claims expressed in this article are solely those of the authors and do not necessarily represent those of their affiliated organizations, or those of the publisher, the editors and the reviewers. Any product that may be evaluated in this article, or claim that may be made by its manufacturer, is not guaranteed or endorsed by the publisher.
