# Supplementary material for: Combination of Anlotinib and Celecoxib for the Treatment of Abdominal Desmoid Tumor: A Case Report and Literature Review
Source: Front Oncol. 2022 Jan 13;11:830672. doi: 10.3389/fonc.2021.830672 (PMC8792388; doi:10.3389/fonc.2021.830672)
Supplement: Supplementary file 1 [file DataSheet_1.docx]

**
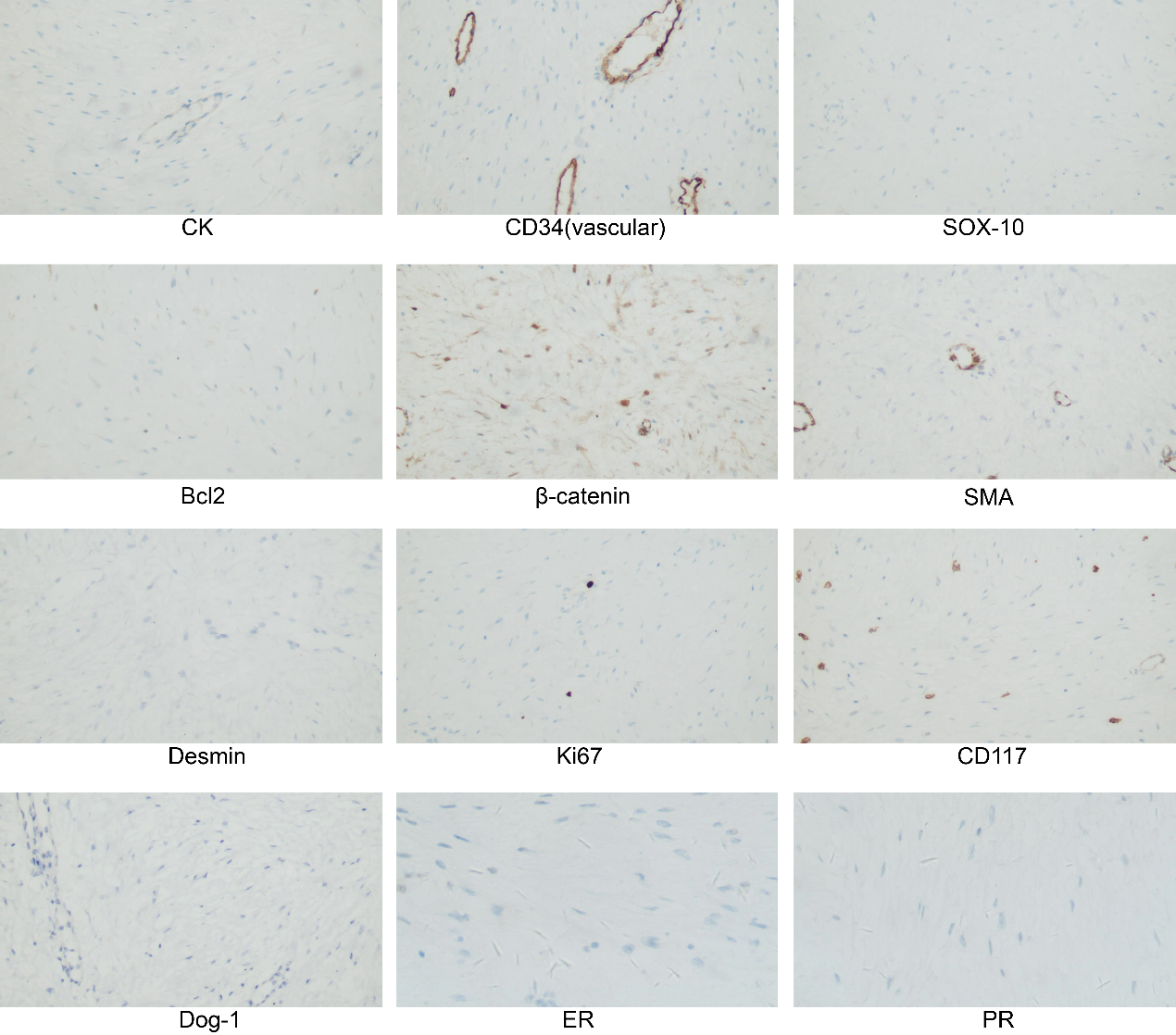
**

**Supplementary Figure1.** **Immunohistochemical indexes of desmoid tumor.** **CK(-), CD34(vascular+), SOX-10(-), Bcl-2 (-),β- catenin(+), SMA(-), Desmin(-) , Ki-67 (about 1%), CD117 (-), Dog-1 (-), ER-, PR-.**
